# Evaluating Prediction Models with Hearing Handicap Inventory for the Elderly in Chronic Otitis Media Patients

**DOI:** 10.3390/diagnostics14182000

**Published:** 2024-09-10

**Authors:** Hee Soo Yoon, Min Jin Kim, Kang Hyeon Lim, Min Suk Kim, Byung Jae Kang, Yoon Chan Rah, June Choi

**Affiliations:** 1 Department of Otorhinolaryngology-Head and Neck Surgery, Korea University College of Medicine, Ansan Hospital, Ansan 15355, Republic of Korea; yhsjoa@hanmail.net (H.S.Y.); kingsonl@hanmail.net (K.H.L.); alexeric@naver.com (M.S.K.); wiagdg@gmail.com (B.J.K.); ycrah@naver.com (Y.C.R.); 2 Department of Biostatistics, Korea University College of Medicine, Seoul 08308, Republic of Korea; aron0129@naver.com; 3 Biomedical Research Center, Korea University Ansan Hospital, Ansan 15355, Republic of Korea; 4 Department of Biomedical Informatics, College of Medicine, Korea University, Seoul 02841, Republic of Korea

**Keywords:** HHIE, hearing prediction, chronic otitis media, hearing level

## Abstract

Background: This retrospective, cross-sectional study aimed to assess the functional hearing capacity of individuals with Chronic Otitis Media (COM) using prediction modeling techniques and the Hearing Handicap Inventory for the Elderly (HHIE) questionnaire. This study investigated the potential of predictive models to identify hearing levels in patients with COM. Methods: We comprehensively examined 289 individuals diagnosed with COM, of whom 136 reported tinnitus and 143 did not. This study involved a detailed analysis of various patient characteristics and HHIE questionnaire results. Logistic and Random Forest models were employed and compared based on key performance metrics. Results: The logistic model demonstrated a slightly higher accuracy (73.56%), area under the curve (AUC; 0.73), Kappa value (0.45), and F1 score (0.78) than the Random Forest model. These findings suggest the superior predictive performance of the logistic model in identifying hearing levels in patients with COM. Conclusions: Although the AUC for the logistic regression did not meet the benchmark, this study highlights the potential for enhanced reliability and improved performance metrics using a larger dataset. The integration of prediction modeling techniques and the HHIE questionnaire shows promise for achieving greater diagnostic accuracy and refining intervention strategies for individuals with COM.

## 1. Introduction

Chronic Otitis Media (COM) presents a significant clinical challenge as precise diagnosis and effective intervention require accurate assessment of functional hearing capacity. In this era of advanced technology and data-driven healthcare, the integration of prediction modeling techniques offers promise for enhancing our understanding of the hearing status of patients with COM. The Hearing Handicap Inventory for the Elderly (HHIE) questionnaire, with its insightful inquiries into the impact of hearing impairment on individuals, is a valuable tool for this assessment [1,2,3,4].

This study attempted to harness the potential of predictive models to elucidate the nuances of the hearing status of patients with COM. Patients with COM may have preconceived notions about their hearing abilities, which can bias their self-assessments. For example, they may believe that their hearing is better than it is because of their desire to avoid acknowledging their hearing problems. Moreover, patients with COM often adapt to their reduced hearing capabilities by adjusting their communication strategies, such as increasing television volumes or asking others to repeat themselves. This adaptation can create a false sense of adequate hearing as they have developed coping mechanisms to manage their condition. Consequently, they may underestimate the extent of hearing loss during the self-assessment.

This study was motivated by the need to provide assistance to COM patients who may be unable to undergo pure-tone audiometry owing to economic, social, or structural constraints. This study explored alternative avenues for individuals facing such limitations using the HHIE questionnaire. Patients who, for various reasons, cannot access traditional diagnostic methods often find themselves excluded from comprehensive hearing assessments. To address this gap, our research contemplates the applicability of the HHIE questionnaire as a viable substitute, offering a practical solution for those for whom economic, social, or structural factors render pure-tone audiometry impractical. Previous studies have suggested a correlation between HHIE and pure-tone threshold average (PTA) [5,6,7]. These studies demonstrate an association between HHIE and hearing ability, proving that HHIE can serve as an objective tool. While previous studies have established the objectivity of HHIE in assessing hearing impairment, our study sought to advance this understanding by testing a new prediction model based on HHIE. This model aims to provide a more nuanced and accurate assessment of hearing status in patients with COM, potentially improving both the diagnostic process and subsequent interventions. By leveraging the strengths of HHIE in conjunction with predictive analytics, this study endeavored to create a robust tool that could bridge the gap for patients who are otherwise unable to access traditional hearing assessments, such as PTA.

Through a meticulous examination of a diverse cohort of individuals diagnosed with COM, we explored not only the quantitative aspects of their hearing but also the qualitative facets of their experiences. Several factors affect the prediction of hearing loss using the questionnaire [8,9,10,11]. We investigated the intricate interplay of variables that influence hearing outcomes, including the presence or absence of tinnitus, demographic characteristics, and the rich tapestry of responses collected through the HHIE questionnaire.

By adopting a multidimensional approach that integrates auditory biomarkers, prediction modeling techniques, and the HHIE questionnaire, this study contributes significantly to the improved assessment of serviceable hearing in individuals with COM. Through this comprehensive framework, this study enriches the understanding of hearing impairment and paves the way for more effective diagnostic and interventional strategies.

## 2. Materials and Methods

### 2.1. Patient Selection and Data Collection

In total, 289 patients with COM who underwent mastoidectomy at Korea University Ansan Hospital between September 2020 and December 2022 were included in this study. All patients had a history of surgery encompassing three types of procedures: open cavity (OC), intact canal wall (ICW), and intact-bridge mastoidectomy (IBM). IBM and OC mastoidectomy differ anatomically in the management of chronic ear diseases. IBM creates a bridge between the mastoid and external auditory canals, preserving part of the posterior canal wall. In contrast, OC completely removes the posterior canal wall, eliminating the external ear canal and establishing a direct opening to the mastoid. While both enhance aeration and drainage, IBM is suitable for less extensive mastoid diseases and maintains external ear canal integrity. OC is chosen when ongoing access and direct observation of the mastoid are crucial because of its more extensive pathology. The choice depends on the specific anatomical needs and the extent of disease in each patient [12,13,14].

Data collection was meticulously conducted using direct preoperative questionnaires and face-to-face interviews with each patient by an attending physician. This approach ensured a comprehensive understanding of the preoperative hearing status of each patient. Additionally, the patients completed the HHIE questionnaire after surgery. This survey contained 10 items that asked subjects to rate their hearing handicaps under specific listening conditions, such as “*When you meet a new friend, will your hearing loss make you embarrassed or uneasy?*” Each rating was scored as follows: 4 = always, 2 = sometimes, and nil for never. A higher total score indicates a greater handicap resulting from hearing loss [15].

Individuals who lacked the ability to comprehend the questionnaire, such as those unable to communicate in Korean, or those with difficulties expressing themselves, were excluded. Additionally, individuals with a history of middle ear or hearing surgery were excluded because they could not undergo hearing tests. Individuals with incomplete postoperative follow-ups were excluded.

Given the critical need to assess the hearing status of patients with COM, especially in regions where PTA may not be readily accessible due to economic or technical limitations, this study aimed to develop a predictive model using the HHIE questionnaire. While PTA remains the gold standard for hearing assessment, our model seeks to serve as a practical alternative for screening patients in resource-limited settings and identifying those who may require more detailed evaluations. Comprehensive data collected from the patients, including their chronic conditions and environmental factors, were integral to this analysis because these variables may significantly influence hearing outcomes.

### 2.2. Outcome and Other Variables

The main objective of this study was to demonstrate the correlation between HHIE and PTA using novel prediction models. Within the dataset, we designated the term “better ear” to signify the ear with a lower threshold between the left and right ears on the PTA. For the analysis, we considered a “significant event (not better ear, hearing loss)” to have occurred if the PTA was over 25 dB (the average of the hearing threshold levels at a set of four frequencies: 500, 1000, 2000, and 3000 Hz); otherwise, we classified it as “normal”. We also provided descriptions of the participants’ characteristics pertaining to their history of smoking, hypertension, diabetes mellitus, hyperlipidemia, stroke, chronic kidney disease, and myocardial infarction.

### 2.3. Statistical Analysis and Modeling

We applied both logistic and Random Forest models, and for each approach, we established metrics including accuracy, area under the curve (AUC), Kappa, and F1 score. These data were subsequently divided into training and test sets in a 7:3 ratio. ‘Accuracy’ illustrates the model’s capability to differentiate between normal and abnormal groups, while AUC offers insight into the model’s ability to predict “significant events”. The Kappa and F1 scores evaluated the extent of agreement between the two groups, highlighting the degree to which similar values were observed by chance. Higher values of accuracy, AUC, and F1 score indicate enhanced performance, whereas a lower Kappa value suggests a comparatively lower agreement. Our initial steps involved the selection of pertinent variables for the baseline model using a stepwise logistic regression method, which aided in identifying significant variables. In the Random Forest model, the selected hyperparameters were the number of trees and the number of variables randomly sampled as candidates for each split. For the logistic regression model, hyperparameter tuning was conducted using penalized maximum likelihood estimation (MLE) to optimize the penalty parameter. After evaluating the coefficient estimates across different values of the tuning parameter λ, we employed 10-fold cross-validation to identify the optimal λ that minimized the mean-square error. Through this process, we conducted a comparison between actual values (labeled as “Reference”) and predicted values (labeled as “Prediction”), leading to the computation of accuracy, AUC, Kappa, and F1 score values for both models. This approach allowed us to comprehensively evaluate the model’s performance and predictive capabilities. R version 4.0.3 (http://www.R-project.org) was used for the statistical analyses.

## 3. Results

### 3.1. Patient Baseline Characteristics

**Figure 1:**
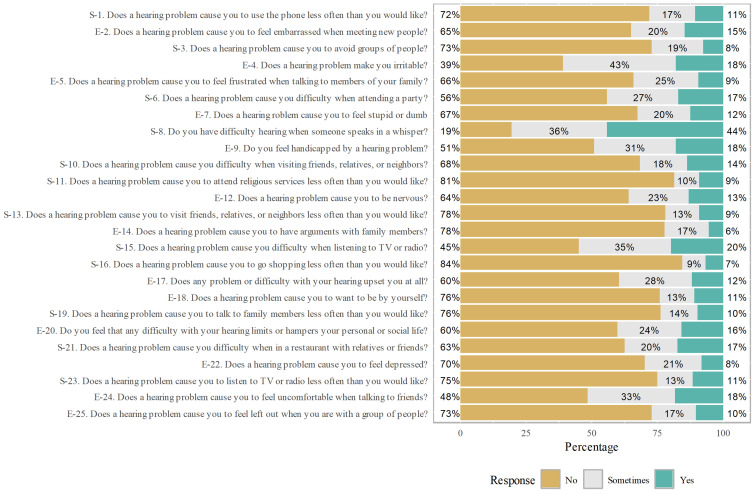
Illustration of the outcomes of the Hearing Handicap Inventory for the Elderly questionnaire in the patient population.

Table 1 presents the fundamental characteristics of patients with COM, stratified based on their hearing levels, categorized as the “better ear” and the “not better ear”. The differentiation criteria between the two categories were determined using PTA levels. An audiometric value exceeding 25 dB was classified as the “not better ear”, while a value below 25 dB was categorized as the “better ear”. The cohort comprised 111 and 178 patients by hearing level, respectively. Notably, a greater percentage of patients did not report hypertension, diabetes mellitus, hyperlipidemia, stroke, or chronic kidney disease between the two groups. Figure 1 illustrates the results derived from the HHIE questionnaire used in this study. Notably, this figure highlights that among the questionnaire items, question E-4—“Does a hearing problem make you irritable?”—garnered a notably higher percentage of ‘yes’ responses than the other questions. Similarly, question S-8—“Do you have difficulty hearing when someone speaks in a whisper?”—elicited a greater proportion of “sometimes” responses compared to the other questions.

### 3.2. Comparison of the Logistic Model and Random Forest Model

Prior to model fitting, pertinent variables were selected from the HHIE questionnaire. Using stepwise logistic regression, we identified significant variables, as summarized in Table 2.

**Figure 2:**
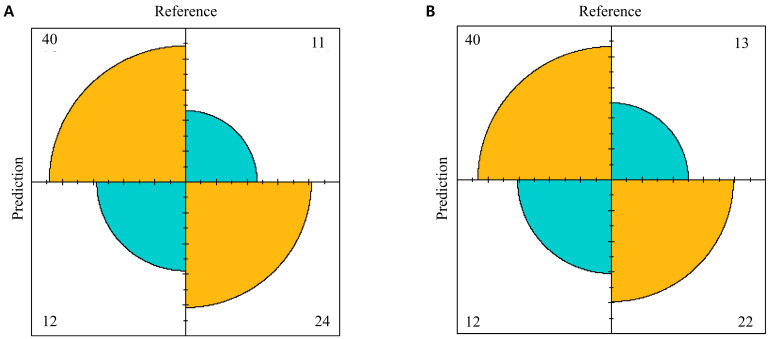
The confusion matrices of the logistic and Random Forest models for the test set. (**A**) The logistic model showcased its prowess through successful predictions. (**B**) The Random Forest model exhibited predictive capability through accurate predictions.

The confusion matrices of the logistic and Random Forest models for the test set are shown (Figure 2). When conducting a comparison between the actual values, referred to as “Reference”, and the anticipated values, denoted as “Predicted”, the logistic model showcased its prowess by successfully predicting 24 out of 36 events (Figure 2A). In contrast, the Random Forest model accurately predicted 22 of the 34 events (Figure 2B).

In the Random Forest method, we used the number of trees and variables randomly sampled as candidates for each split as the hyperparameters. Although the default number of trees is typically set to 500, we reduced this to 100 because of the small sample size to prevent overfitting. For the number of variables randomly sampled at each split, we used the square root of the total number of variables, resulting in three variables being used at each split. The Out-of-Bag (OOB) estimate of the error rate was 24.88%.

**Figure 3:**
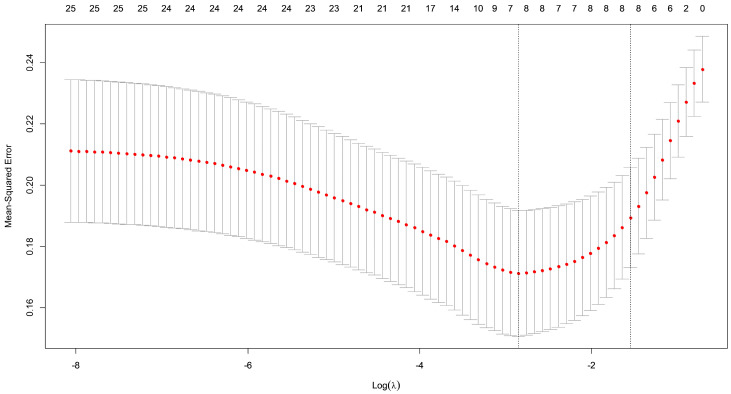
Mean-Squared Error plot according to log after cross-validation in logistic regression. This plot illustrates that the log(λ) around −3.45 provided the smallest mean-square error.

For the logistic regression model, we optimized the hyperparameters using penalized MLE to adjust the penalty. After evaluating the coefficient estimates across different values of the tuning parameter λ, we conducted 10-fold cross-validation to determine the optimal λ that minimized the mean-square error. The log(λ) value of approximately −3.45 yielded the smallest error, and this value was subsequently used to fit the model and evaluate the performances. This is shown in Figure 3.

A comprehensive overview of the statistical metrics for the logistic and the Random Forest models is presented (Table 3). This table summarizes the performance of each model using key indicators, including accuracy, AUC, Kappa, and F1 scores.

**Figure 4:**
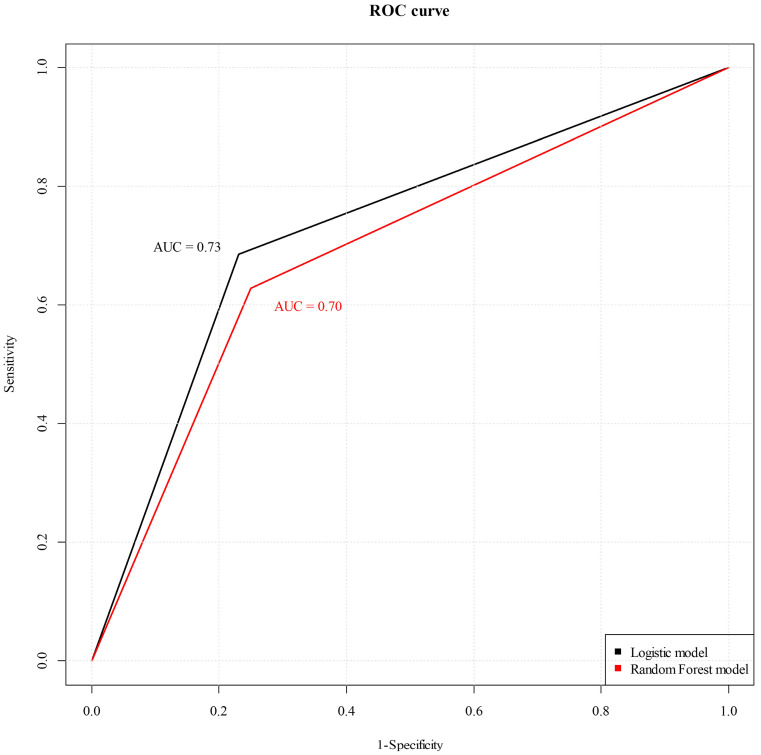
The ROC curve of the logistic model and the Random Forest model. In the logistic model, the AUC was 0.73, indicating the capability of the model to discriminate between positive and negative cases. In contrast, the Random Forest model showed that the AUC was 0.70, indicating commendable discrimination ability.

The ROC curves of the logistic and Random Forest models are shown (Figure 4). In the logistic model, the accuracy was 73.56% (95% confidence interval [CI]: 63.02–82.45%), signifying the proportion of correctly predicted outcomes. In addition, the AUC was 0.73, indicating the capability of the model to discriminate between positive and negative cases. A Kappa value of 0.45 signified agreement beyond chance, implying a moderate level of concordance between the observed and predicted outcomes. The F1 score, a blend of precision and recall, achieved a value of 0.78, reflecting the balance of the model between accurate positive predictions and the avoidance of false positives.

In contrast, the Random Forest model showed an accuracy of 71.26% (95% CI [60.57–80.46%]), which was slightly lower than that of the logistic model. The AUC was 0.70, indicating commendable discrimination ability. The Kappa value (0.38) suggested a moderate level of agreement, although it was lower than that of the logistic model. The F1 score was 0.75, indicating harmonized performance in terms of precision and recall.

The results indicated that the logistic model demonstrated a slightly higher accuracy, AUC, Kappa value, and F1 score than the Random Forest model. This suggests that the logistic model has a stronger predictive performance, encompassing both its ability to differentiate between classes (AUC) and its agreement measure (Kappa), while also excelling at balancing precision and recall (F1 score), when compared with the Random Forest model.

## 4. Discussion

This study makes a useful contribution to the ongoing exploration of HHIE for predicting hearing levels without audiograms. This innovative approach has promising practical implications, particularly for individuals who face challenges in accessing audiological services, such as those unable to afford visits to ENT clinics or those residing in resource-constrained regions. By harnessing the potential of the HHIE questionnaire to predict hearing levels, we offer a cost-effective and accessible alternative that can aid in the early identification of hearing impairments and guide appropriate interventions. This not only extends the reach of audiological assessments to a wider population, but also underscores the importance of leveraging existing tools and methodologies to address the pressing healthcare needs of diverse communities.

Previous studies have acknowledged that the HHIE questionnaire is a potent tool for assessing the impact of hearing impairment on an individual’s quality of life, underscoring its emotional and social dimensions beyond conventional audiometric measurements [7,16]. Our incorporation of the HHIE questionnaire in this study is part of a broader effort to comprehensively understand the subjective experiences of patients with COM and their connection to objective hearing levels.

Drawing on existing research, we extracted valuable insights into the significance of this study and its implications for clinical practice [17,18]. Our study highlights the intricate relationship between data volume and predictive performance metrics, which is a growing concern in otolaryngology. In recent years, prediction modeling techniques such as machine learning have gained traction for predicting hearing loss across diverse domains. For instance, predictive models have successfully estimated hearing loss among workers exposed to complex industrial noise, as evidenced in prior studies [19]. Furthermore, machine learning models have been developed to predict hearing outcomes in cases of idiopathic sudden sensorineural hearing loss, highlighting the expanding applications of machine learning in audiology [20,21,22]. The application of machine learning in predicting noise-induced hearing loss has also been explored, highlighting its growing relevance to hearing-related research [23]. Recent investigations have focused on predicting hearing outcomes following tympanoplasty, indicating its utility in clinical scenarios [24]. In addition to hearing loss prediction, the potential of prediction modeling techniques such as machine learning for diagnosing ear diseases has gained prominence in recent studies [25,26,27,28,29].

Our study emphasizes the importance of recognizing that in situations with limited data availability, the full potential of predictive performance metrics may remain underutilized. One potential limitation is the reliance on a relatively modest dataset of 289 patients with COM. This study acknowledges the importance of data volume in predictive modeling, and the observed limitations in data availability may affect the full realization of predictive performance metrics. A larger and more diverse dataset could enhance the accuracy and reliability of the predictive model, providing a more robust foundation for the integration of simple machine learning techniques with the HHIE questionnaire. Additionally, the study acknowledges the potential of dataset augmentation to address this limitation, but the extent to which this can be achieved and its impact on model improvement remain areas for further exploration and consideration.

The focal point of our research, the relationship between data volume and predictive performance metrics, aligns with earlier findings, highlighting the critical role of robust datasets in predictive modeling. Our study reinforces the need for larger and more diverse datasets to fully reveal the predictive potential of prediction models using simple machine learning techniques, aligning with previous research demonstrating the value of extensive data in enhancing the accuracy and reliability of predictive models.

## 5. Conclusions

Our study introduces a pioneering approach to predict hearing levels in patients with COM using the HHIE questionnaire and machine learning techniques. The integration of the HHIE with logistic regression and Random Forest models revealed notable findings regarding the predictive performance of hearing outcomes.

The results highlight that the logistic regression model outperformed the Random Forest model across key performance indicators, including accuracy, AUC, Kappa, and F1 score. This suggests that the logistic regression model is more effective in predicting hearing levels, balancing precision and recall, and achieving a higher agreement between the observed and predicted outcomes.

Although the logistic regression model’s AUC fell slightly short of the desired benchmark, future efforts to expand and diversify the dataset present opportunities to further enhance the model’s performance. This advancement may lead to more reliable predictive capabilities and effective clinical assessment tools for patients with COM.

However, it is important to acknowledge the potential biases inherent in our approach. The selection bias introduced by the specific patient population may limit the generalizability of our findings. Additionally, the use of self-reported data from the HHIE questionnaire introduces the risk of measurement bias, which could affect the accuracy of our predictions. Furthermore, model bias stemming from the assumptions of the logistic regression model and complexity of the Random Forest model may have affected the interpretability and reliability of the results. These biases underscore the need for a cautious interpretation of our findings and highlight the importance of future research to mitigate these limitations. By expanding our dataset, incorporating more diverse patient populations, and exploring advanced modeling techniques, we can further enhance the robustness and applicability of our predictive models. As we continue to explore the untapped potential of the HHIE, addressing these biases will be critical for developing a more inclusive and effective approach to hearing level assessment. This will ultimately benefit individuals globally and foster greater equity in healthcare access, paving the way for a more precise and comprehensive assessment of hearing in patients with COM.

In summary, our study successfully illustrated the potential of the HHIE questionnaire combined with machine learning techniques to predict hearing levels without relying on traditional audiograms. These results highlight the innovative nature of our approach and its potential for advancing the field of otology. Continued exploration and refinement of this method will contribute to more inclusive and effective hearing assessments, benefiting patients globally and promoting greater equity in healthcare access.

## Figures and Tables

**Table 1:** Baseline characteristics of the study population stratified by hearing level.

Characteristics	Level	Not Better Ear (>25 dB)	Better Ear (≤25 dB)	SMD
Total Number		111	178	
Age (mean (SD))		61.81 (10.52)	49.31 (15.36)	0.95
Height (mean (SD))		159.29 (8.73)	163.73 (9.09)	0.498
Weight (mean (SD))		62.54 (10.65)	65.44 (14.09)	0.232
BMI (mean (SD))		24.60 (3.53)	24.28 (4.18)	0.083
Sex (%)	Female	63 (56.8)	98 (55.1)	0.034
	Male	48 (43.2)	80 (44.9)	
Surgery Method	OC	49 (44.1)	64 (36.0)	0.219
	ICW	59 (53.2)	105 (59.0)	
	IBM	1 (0.9)	1 (0.6)	
	Other	2 (1.8)	8 (4.5)	
Surgery Direction (%)	Left	56 (50.5)	87 (48.9)	0.031
	Right	55 (49.5)	91 (51.1)	
Tinnitus (%)	No	46 (41.4)	97 (54.5)	0.264
	Yes	65 (58.6)	81 (45.5)	
Tinnitus Location (%)	Both	16 (14.4)	7 (3.9)	0.42
	Left	27 (24.3)	35 (19.7)	
	None	46 (41.4)	98 (55.1)	
	Right	22 (19.8)	38 (21.3)	
Smoker (%)	Current Smoker	15 (13.5)	33 (18.5)	0.255
	Non-smoker	87 (78.4)	120 (67.4)	
	Ex-smoker	9 (8.1)	25 (14.0)	
HTN (%)	Yes	46 (41.4)	38 (21.3)	0.443
	No	65 (58.6)	140 (78.7)	
DM (%)	Yes	10 (9.0)	21 (11.8)	0.091
	No	101 (91.0)	157 (88.2)	
Hyperlipidemia (%)	Yes	11 (9.9)	30 (16.9)	0.205
	No	100 (90.1)	148 (83.1)	
Stroke (%)	Yes	2 (1.8)	0 (0.0)	0.192
	No	109 (98.2)	178 (100.0)	
CKD (%)	Yes	2 (1.8)	3 (1.7)	0.009
	No	109 (98.2)	175 (98.3)	

Body Mass Index, BMI; hypertension, HTN; diabetes mellitus, DM; chronic kidney disease, CKD. Standard mean deviation, SMD.

**Table 2:** Significant questionnaire items of Hearing Handicap Inventory for the Elderly (HHIE).

HHIE Questionnaires
E-4. Does a hearing problem make you irritable?
E-5. Does a hearing problem cause you to feel frustrated when talking to members of your family?
E-14. Does a hearing problem cause you to have arguments with family members?
E-24. Does a hearing problem cause you to feel uncomfortable when talking to friends?
E-25. Does a hearing problem cause you to feel left out when you are with a group of people?
S-6. Does a hearing problem cause you difficulty when attending a party?
S-8. Do you have difficulty hearing when someone speaks in a whisper?
S-11. Does a hearing problem cause you to attend religious services less often than you would like?
S-15. Does a hearing problem cause you difficulty when listening to TV or radio?
S-16. Does a hearing problem cause you to go shopping less often than you would like?
S-21. Does a hearing problem cause you difficulty when in a restaurant with relatives or friends?
S-23. Does a hearing problem cause you to listen to TV or radio less often than you would like?

**Table 3:** Statistical parameters of the logistic model and Random Forest model.

Model	Accuracy (95%CI)	Sensitivity (%)	Specificity (%)	AUC	Kappa	F1
Logistic	73.56% (63.02–82.45%)	76.92%	68.57%	0.73	0.45	0.78
Random Forest	71.26% (60.57–80.46%)	75.00%	62.85%	0.70	0.38	0.75

## Data Availability

The dataset used in this study is not publicly available. However, the data in this study can be obtained upon reasonable request from the corresponding author.

## References

[1] Weinstein B.E., Spitzer J.B., Ventry I.M. (1986). Test-retest reliability of the Hearing Handicap Inventory for the Elderly. Ear Hear..

[2] Newman C.W., Weinstein B.E. (1989). Test-retest reliability of the Hearing Handicap Inventory for the Elderly using two administration approaches. Ear Hear..

[3] Lichtenstein M.J., Bess F.H., Logan S.A. (1988). Validation of screening tools for identifying hearing-impaired elderly in primary care. JAMA.

[4] Whittemore K.R., Merchant S.N., Rosowski J.J. (1998). Acoustic mechanisms: Canal wall-up versus canal wall-down mastoidectomy. Otolaryngol. Head Neck Surg..

[5] Camarudin N., Ahmad S.A., Minhat H.S., Mohamed M.H., Adnan R.N.E.R. (2022). Correlation between Hearing Handicap Inventory for the Elderly Screening (HHIE-S) and Pure Tone Audiometry (PTA) Test among Malaysian Elderly. Malays. J. Med. Health Sci..

[6] Wang Y., Mo L., Li Y., Zheng Z., Qi Y. (2017). Analysing use of the Chinese HHIE-S for hearing screening of elderly in a northeastern industrial area of China. Int. J. Audiol..

[7] Servidoni A.B., Conterno L.O. (2018). Hearing Loss in the Elderly: Is the Hearing Handicap Inventory for the Elderly—Screening Version Effective in Diagnosis When Compared to the Audiometric Test?. Int. Arch. Otorhinolaryngol..

[8] McBride D. (1993). Hearing conservation in the mining industry. Evaluation of a risk factor questionnaire. Occup. Med..

[9] Saunders G.H., Cienkowski K.M. (1996). Refinement and psychometric evaluation of the Attitudes Toward Loss of Hearing Questionnaire. Ear Hear..

[10] Burr H., Lund S.P., Sperling B.B., Kristensen T.S., Poulsen O.M. (2005). Smoking and height as risk factors for prevalence and 5-year incidence of hearing loss. A questionnaire-based follow-up study of employees in Denmark aged 18–59 years exposed and unexposed to noise. Int. J. Audiol..

[11] Demeester K., Topsakal V., Hendrickx J.J., Fransen E., van Laer L., Van Camp G., Van de Heyning P., van Wieringen A. (2012). Hearing disability measured by the speech, spatial, and qualities of hearing scale in clinically normal-hearing and hearing-impaired middle-aged persons, and disability screening by means of a reduced SSQ (the SSQ5). Ear Hear..

[12] Bennett M., Warren F., Haynes D. (2006). Indications and technique in mastoidectomy. Otolaryngol. Clin. N. Am..

[13] Haynes D.S. (2001). Surgery for chronic ear disease. Ear Nose Throat J..

[14] Sheehy J.L. (1994). Mastoidectomy: The Intact Canal Wall Procedure. Otologic Surgery.

[15] Lichtenstein M.J., Bess F.H., Logan S.A. (1988). Diagnostic performance of the hearing handicap inventory for the elderly (screening version) against differing definitions of hearing loss. Ear Hear..

[16] Tomioka K., Ikeda H., Hanaie K., Morikawa M., Iwamoto J., Okamoto N., Saeki K., Kurumatani N. (2013). The Hearing Handicap Inventory for Elderly-Screening (HHIE-S) versus a single question: Reliability, validity, and relations with quality of life measures in the elderly community, Japan. Qual. Life Res..

[17] Newman C.W., Weinstein B.E. (1988). The Hearing Handicap Inventory for the Elderly as a measure of hearing aid benefit. Ear Hear..

[18] Newman C.W., Weinstein B.E., Jacobson G.P., Hug G.A. (1990). The Hearing Handicap Inventory for Adults: Psychometric adequacy and audiometric correlates. Ear Hear..

[19] Zhao Y., Li J., Zhang M., Lu Y., Xie H., Tian Y., Qiu W. (2019). Machine Learning Models for the Hearing Impairment Prediction in Workers Exposed to Complex Industrial Noise: A Pilot Study. Ear Hear..

[20] Park K.V., Oh K.H., Jeong Y.J., Rhee J., Han M.S., Han S.W., Choi J. (2020). Machine Learning Models for Predicting Hearing Prognosis in Unilateral Idiopathic Sudden Sensorineural Hearing Loss. Clin. Exp. Otorhinolaryngol..

[21] Bing D., Ying J., Miao J., Lan L., Wang D., Zhao L., Yin Z., Yu L., Guan J., Wang Q. (2018). Predicting the hearing outcome in sudden sensorineural hearing loss via machine learning models. Clin. Otolaryngol..

[22] Uhm T., Lee J.E., Yi S., Choi S.W., Oh S.J., Kong S.K., Lee I.W., Lee H.M. (2021). Predicting hearing recovery following treatment of idiopathic sudden sensorineural hearing loss with machine learning models. Am. J. Otolaryngol..

[23] Chen F., Cao Z., Grais E.M., Zhao F. (2021). Contributions and limitations of using machine learning to predict noise-induced hearing loss. Int. Arch. Occup. Environ. Health.

[24] Koyama H., Kashio A., Uranaka T., Matsumoto Y., Yamasoba T. (2023). Application of Machine Learning to Predict Hearing Outcomes of Tympanoplasty. Laryngoscope.

[25] Crowson M.G., Hartnick C.J., Diercks G.R., Gallagher T.Q., Fracchia M.S., Setlur J., Cohen M.S. (2021). Machine Learning for Accurate Intraoperative Pediatric Middle Ear Effusion Diagnosis. Pediatrics.

[26] Wang Y.M., Li Y., Cheng Y.S., He Z.Y., Yang J.M., Xu J.H., Chi Z.C., Chi F.L., Ren D.D. (2020). Deep Learning in Automated Region Proposal and Diagnosis of Chronic Otitis Media Based on Computed Tomography. Ear Hear..

[27] Zeng J., Kang W., Chen S., Lin Y., Deng W., Wang Y., Chen G., Ma K., Zhao F., Zheng Y. (2022). A Deep Learning Approach to Predict Conductive Hearing Loss in Patients with Otitis Media with Effusion Using Otoscopic Images. JAMA Otolaryngol. Head Neck Surg..

[28] Byun H., Yu S., Oh J., Bae J., Yoon M.S., Lee S.H., Chung J.H., Kim T.H. (2021). An Assistive Role of a Machine Learning Network in Diagnosis of Middle Ear Diseases. J. Clin. Med..

[29] Viscaino M., Maass J.C., Delano P.H., Torrente M., Stott C., Auat Cheein F. (2020). Computer-aided diagnosis of external and middle ear conditions: A machine learning approach. PLoS ONE.

